# Corrigendum to “Determination of thymic function directly from peripheral blood: A validated modification to an established method” [J. Immunol. Methods 2008 339(2):185–94]

**DOI:** 10.1016/j.jim.2009.03.016

**Published:** 2009-05-15

**Authors:** A.R. Lorenzi, A.M. Patterson, A. Pratt, M. Jefferson, C.E. Chapman, F. Ponchel, J.D. Isaacs

**Affiliations:** a Musculoskeletal Research Group, Institute of Cellular Medicine, Newcastle University, Newcastle-upon-Tyne, NE2 4HH, UK; b Gut Immunology, Gut Health Division, Rowett Research Group, Greenburn Road, Aberdeen, AB21 9SB, UK; c NBS, Newcastle Centre, Holland Drive, Barrack Road, Newcastle-upon-Tyne NE2 4NQ, UK; d Leeds Institute of Molecular Medicine, Section of Musculoskeletal Disease, Leeds University, Leeds, UK

The authors regret that an error occurred in Section 2.4. Real time quantitative PCR, on the fifth sentence.

The corrected sentence is 'Each reaction contained 200 ng DNA.'

